# Curcumin, Quercetin, Catechins and Metabolic Diseases: The Role of Gut Microbiota

**DOI:** 10.3390/nu13010206

**Published:** 2021-01-12

**Authors:** Umair Shabbir, Momna Rubab, Eric Banan-Mwine Daliri, Ramachandran Chelliah, Ahsan Javed, Deog-Hwan Oh

**Affiliations:** 1 Department of Food Science and Biotechnology, College of Agriculture and Life Sciences, Kangwon National University, Chuncheon 24341, Korea; umair336@gmail.com (U.S.); rubab.momna@gmail.com (M.R.); ericdaliri@kangwon.ac.kr (E.B.-M.D.); ramachandran865@gmail.com (R.C.); 2 Department of Food Science & Biotechnology, College of Agriculture and Life Sciences, Kyungpook National University, Daegu 41566, Korea; ahsanjaved@knu.ac.kr

**Keywords:** plant polyphenols, metabolic syndrome, biotransformation, bioavailability, gut health

## Abstract

Polyphenols (PPs) are the naturally occurring bioactive components in fruits and vegetables, and they are the most abundant antioxidant in the human diet. Studies are suggesting that ingestion of PPs might be helpful to ameliorate metabolic syndromes that may contribute in the prevention of several chronic disorders like diabetes, obesity, hypertension, and colon cancer. PPs have structural diversity which impacts their bioavailability as they accumulate in the large intestine and are extensively metabolized through gut microbiota (GM). Intestinal microbiota transforms PPs into their metabolites to make them bioactive. Interestingly, not only GM act on PPs to metabolize them but PPs also modulate the composition of GM. Thus, change in GM from pathogenic to beneficial ones may be helpful to ameliorate gut health and associated diseases. However, to overcome the low bioavailability of PPs, various approaches have been developed to improve their solubility and transportation through the gut. In this review, we present evidence supporting the structural changes that occur after metabolic reactions in PPs (curcumin, quercetin, and catechins) and their effect on GM composition that leads to improving overall gut health and helping to ameliorate metabolic disorders.

## 1. Introduction

Plant polyphenols (PPs) are secondary metabolites and are ubiquitously found in various parts of plants like roots, stems, leaves, flowers, and pulp. PPs are considered to be very necessary for plant survival in the environment but not directly responsible for the development and growth of plants. PPs are produced from primary metabolites and intermediates through unique biosynthetic pathways [1]. They are a class of non-essential phytonutrients and are abundant in fruits, cereals, and vegetables [2,3]. Molecules of PPs display at least one aromatic ring carrying one or more hydroxyl groups [4] and they are also found as conjugate with organic acids or sugars or as polymers (flavonoids). Additionally, PPs are hydrolyzable and condensed tannins represent a special group that interacts with proteins [5]. According to Costa et al. [6], around 8000 polyphenolic structures have been recognized. They are subdivided according to their chemical structure (depending on the number of hydroxyls in the molecule and on the nature and the position of other substituents) into the following structural classes: lignans, phenolic acids, stilbenes, condensed or hydrolyzable tannins, and flavonoids (containing anthocyanins and isoflavonoids). It has been documented that PPs are primarily distributed as glycosides in plants and their elementary structure is aglycones. However, the phenolic acids comprise hydroxycinnamic acids (caffeic, ferulic, chlorogenic acid, p-coumaric, and sinapic) and hydroxybenzoic (gallic, syringic acid, vanillic, and protocatechuic acid) in the non-flavonoid group. Phenolic acids exist in plants as soluble-free, conjugate, and insoluble bound forms. Flavonoids are originated from the acetate/malonate pathways that are stored as glycosides in plants. They constitute a large group of phenolic compounds [7]. The majority of flavonoids contain diphenyl propane which is a closed pyran—a variation in the central pyran ring that is due to oxidation and hydroxylation pattern [8]. The presence of conjugated chromophore is considered to be responsible for yellow and red color development in flavonoids. Anthocyanidins (like cyanidin) are an example which illustrates red and magenta color [9]. Flavonoids and their subgroups (flavones, flavanones, flavonols, and flavanonols) are ubiquitous in the plant kingdom [8]. Other PPs such as lignans (e.g., lariciresinol and pinoresinol) are non-flavonoid diphenolic components that are composed of phenylpropanoid units. Stilbenes represents non-flavonoids (including trans-resveratrol), abundantly present in grapes and red wine [10]. Tannins are further subdivided into two groups: (1) Hydrolysed tannins are the esters of gallic acid and egalic acids, while (2) condensed tannins are the polymers of catechin and epicatechin [8]. The aim of this review is to provide an overview of the biological role of PPs against metabolic syndromes to improve gut health, and their extraction techniques. Specifically, we discussed the role of curcumin, quercetin, and catechins in the gut with their biotransformation into metabolites through gut microbiota (GM) and changes that occur after that.

## 2. Biological Role of PPs

**Figure 1:**
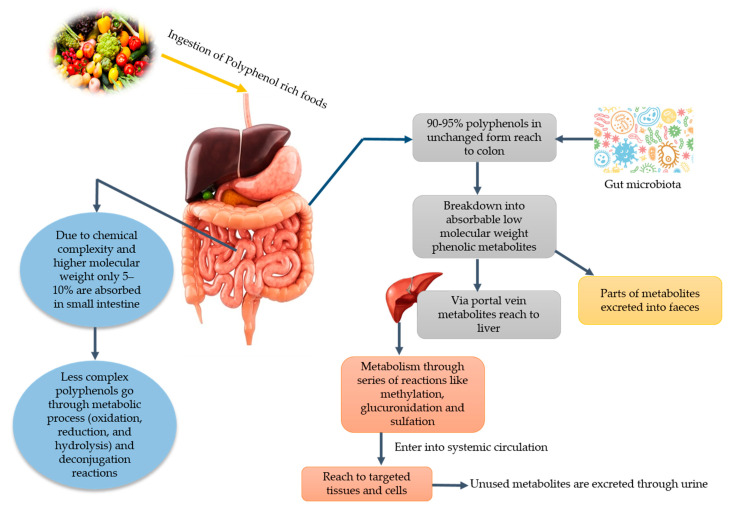
Major dietary sources of polyphenols and potential gut microbiota-associated benefits.

It has been stated that PPs play a vital role in human nutrition as they have antioxidative ability and can decrease the reactive oxygen species (ROS). Moreover, they can be utilized to ameliorate metabolic disorders like obesity, diabetes, cancer, and cardio-metabolic diseases [11]. Among PPs, phenolic acids can lower the risk of chronic diseases like cardiovascular disease, cancer, etc. Since various diseases have been associated with oxidative stress, dietary PPs reduce the effects caused by excessive ROS or other nitrogen species. By neutralizing the free radicals via donating an electron and as direct radical scavengers of the lipid peroxidation chain reactions, PPs eradicate the oxidative stress [12]. Phenolic acids and flavonoids, including anthocyanins, procyanidins, quercetin, catechins, curcumin, and ellagic acids, have their role in obesity and weight management [13]. Despite their role in disease management, PPs lose their therapeutic properties due to glycosylation and become less available to the target cell. Due to the glycosylated form, their absorption mainly takes place in the colon. Gowd et al. [14] briefly documented the metabolism of PPs in humans and reported that PPs have high molecular weight and complex structure; that is why only 5–10% are absorbed in the small intestine. About 90–95% reach the colon and their GM play a significant role in the breaking down of these complexes and converting them into absorbable metabolites. After conversion into phenolic metabolites, they reach the liver through a portal vein upon absorption. Additionally, they undergo extensive degradation via metabolic reactions to form active metabolites. Then, these metabolites enter into systematic circulation reach target cells and tissues where they can show physiological significance. Unused and remaining metabolites are excreted through urine. The schematic ingestion, digestion, absorption, and excretion of PPs are exhibited in Figure 1.

## 3. Extraction of PPs

Although 90–95% of PPs are absorbed in the colon, however, their efficacy (in terms of its therapeutic effects) is about 15–20%. Extraction of PPs is a crucial step, and various techniques can be employed that can help to enhance the bioactivity and bioavailability of extracted PPs. These extracted PPs can be used for many medical and pharmaceutical purposes to utilize their therapeutic values effectively. Being hydrophilic and phenolic in nature, they can be extracted by various solvents, including methanol, acetone, acetonitrile, and ethanol. Among these solvents, methanol is found to be more efficient for the extraction of lower molecular weight PPs while aqueous acetone—for higher molecular weight PPs [15]. Different extraction techniques are utilized for PPs including maceration, heat-assisted, ultrasound-assisted extraction (UAE), microwave-assisted extraction (MAE), homogeniser-assisted extraction (HAE), and rapid solid–liquid dynamic extraction (RSLDE). Maceration is a simple and traditional method of extraction that is performed in glass containers at room temperature [16,17]. Heat-assisted extraction is carried out at a high temperature in glass containers with continuous mixing in a water bath or the incubator shaker, allowing unattended operation in a temperature-controlled environment. The use of thermal energy improves the efficiency of the extraction by disruption of cellular structures, the increment of cell membrane permeability, and breakdown of PPs–lipoprotein interactions, which cause enhancement of PPs solubility and mass transfer [18]. Continuous mixing for minutes or hours is required and the choice of extraction solvent in maceration and heat-assisted extraction depends on the chemical and physical properties of the targeted compound and extraction method. MAE is performed with a microwave extractor system that is equipped with a digital control system. Procedure time (1–25 min), stirring (250 rpm), and temperature (20–50 °C) are used. UAE is carried out in an ultrasound bath or probe and plant tissues are destroyed through ultrasonic waves, as they cause mechanical vibrations that lead to expansion and compression cycles during movement through the extraction medium and provoke the rise of temperature and negative pressure [19,20]. These mechanical and thermal effects cause the degradation of cell walls, the release of cell contents, greater penetration of solvent into plant material, the increment of mass transfer, and thus, the increase of PPs yield [21]. HAE is usually operated at high speed which requires homogenization and centrifugation in the presence of a solvent. The high-shear rate applied promotes the rapid rupture of the plant material, releasing the constituents into the extraction solvent [22]. Besides, RSLDE uses the Naviglio extractor generation in which a suitable solvent is used under the pressure gradient between the outside and the inside of a solid matrix containing extractable material, followed by a sudden restoration of the initial equilibrium conditions, induces forced extraction [23]. Recently, Yilmaz et al. [19] reported a study for the extraction of PPs using maceration, MAE, and UAE methods from *Stevia rebaudiana* Bertoni. In that study, MAE and UAE required less processing time and showed a higher yield as compared to maceration [19]. Galan et al. [24] carried out a comparison study between MAE and conventional health-assisted extraction; they stated that MAE possessed higher yield and antioxidant activity for PPs. Another comparison study among maceration, HAE, UAE, MAE, and RSLDE was conducted and revealed that better extraction was found with 100% methanol as an extraction solvent than methanol/water (50:50, *v*/*v*). The highest phenolic content was found with HAE, followed by UAE then MAE. In another similar study, da Rosa et al. [25] compared MAE, UAE, and maceration and reported that MAE is more efficient in yield with short extraction time, followed by UAE and maceration. Additionally, regarding in vitro antioxidant activity: ferric reducing antioxidant power was found highest with UAE in which water and methanol are used as a solvent. In contrast, oxygen radical absorbance capacity was found highest with HAE with 100% methanol as solvent extraction [26]. Jovanović et al. [27] stated a comparison of extraction techniques and compiled that both maceration and heat-assisted extraction are simple and traditional methods. However, the usage of high solvent in these techniques demands high cost and may lead to environmental problems, whereas extracted PPs yield is also low. On the other hand, UAE and MAE require less time and less solvent. Moreover, they show a higher yield with less environmental issues. From the above-mentioned data, it can be concluded that the selection of the right extraction technique is essential for wider and correct compound characterization. All of the extraction techniques promote the recovery of phenolic compounds but with different efficiencies. So, the use of non-conventional (other than maceration and heat-associated) extraction technologies is suggested.

## 4. Metabolic Syndrome

Hippocrates has been quoted as saying “death sits in the bowels” and “bad digestion is the root of all evil” in 400 B.C., emphasizing on the importance of the diet and human intestines in health and disease, which was recognized long ago [28]. On the other hand, gut metabolic syndromes are multiple risk factors like dyslipidemia, hyperglycemia, oxidative stress, insulin resistance, hypertension, fatty liver, etc. These syndromes result in metabolic diseases such as diabetes, obesity, hepatopathy, nephropathy, inflammation, cardiomyopathy, neurodegeneration, and osteoarthritis [29,30,31]. Metabolic syndromes are one of the concerning issues in the world as their prevalence is increasing day by day. It has been estimated that their prevalence will increase by up to 53% by the year 2035 [32]. Human intestines comprise an intricate ecological colony of dwelling bacteria, known as GM [33]. GM is the colony of collective microbes (mainly bacteria) residing in the gastrointestinal tract (GIT) and about 100 trillion microorganisms live in the human gut [28]. Other than bacteria, protozoa, virus, and eukaryotic organisms, including fungi, are also inhabiting but in a minimal number. The small intestine (duodenum, jejunum, and ileum) comprises several bacteria ranging from 10^4^ bacteria/mL content to 10^6^–10^7^ bacteria/mL at the ileocecal junction. On the other hand, most of the non-sporing anaerobes reside in the large intestine and it has a number of bacteria ranging between 10^11^ and 10^12^/g [34]. On average, 90% of the bacteria in the gut of an adult are phyla Firmicutes (Gram-positive) and phyla Bacteroidetes (Gram-negative); many others are also present but in much lower abundance, such as Verrucomicrobia (Gram-negative), namely *Akkermansia muciniphila* (Gram-negative), and Actinobacteria (Gram-positive), namely *Bifidobacterium,* Proteobacteria (Gram-negative) [35]. GM play a major role in metabolic health (digestion and metabolism), vitamin synthesis, maintains gut homeostasis (a balance of host responses to the beneficial enteric microbial community and the pathogenic stimuli that can arise [36]) and, when aberrant, to the pathogenesis of various common metabolic disorders [37,38]. Further, GM regulate the gut endocrine function and neurological signaling, host immunity, modify drug action and metabolism, eradicate toxins, and produce various composites that affect the host [39]. The qualitative composition of the GM differs, depending on age and eating patterns, e.g., *Bifidobacteria,* and Proteobacteria are found abundantly in the gut of breastfed infants [40]. However, *Bacteroides* and Clostridia prevail abundantly in babies fed with formula milk [41]. While during weaning, Bacteroidetes and Firmicutes compositions seem to increase with the decrease in Proteobacteria and Actinobacteria compositions, conversely, in elders, Bacteroidetes and Proteobacteria tend to upsurge [42].

Moreover, Kumar et al. [33] stated that bacteria in amniotic fluid, genetic background, breastfeeding, solid foods, adulthood dietary habits, aging, exercise, stress, drug (such as antibiotics), and xenobiotics are the major modulators of GM in humans. These modulators are responsible for an impairment in GM composition or function that is known as GM dysbiosis (GMD) [43]. Many studies evidently stated that there is an association between diseases and GMD, including those of the GI tract, such as ulcerative colitis, inflammatory disease, colorectal cancer, obesity, type 2 diabetes, metabolic liver disease, cardiometabolic diseases, Alzheimer’s disease, and Parkinson’s [38,42,44,45,46]. Therefore, researchers are trying to find nutraceutical or therapeutic interventions to develop a healthy GM equilibrium to retard the harmful bacteria and pathobionts without affecting the beneficial or symbionts ones.

## 5. GM and PPs

**Figure 2:**
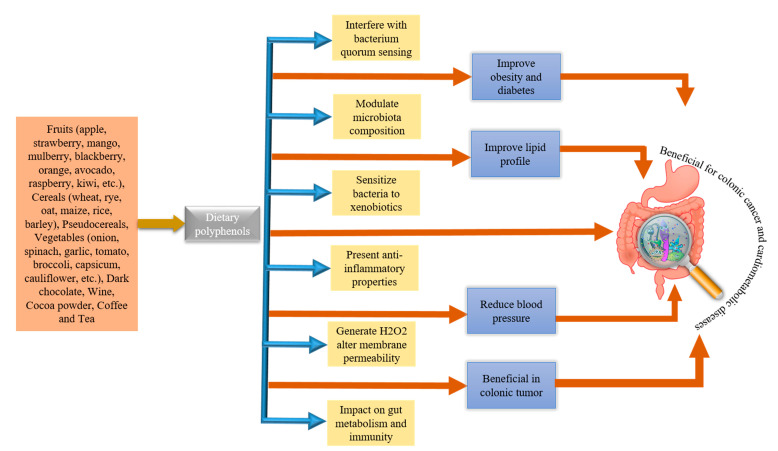
Major dietary sources of polyphenols and potential gut microbiota-associated benefits.

Studies are suggesting that complex and dynamic interplay occurs between PPs and GM during metabolism, contributing a lot to the overall health of individuals. Thus, retention of PPs in intestines for a long time can promote beneficial effect on GM. On the other hand, GM enhance the biological activity of PPs by biotransforming them into active metabolites (phenolics) [37]. Bacterial species, such *Bifidobacterium* sp., *Lactobacillus* sp., *Escherichia coli*, *Bacteroides* sp., *Eubacterium* sp., *Enterococcus caccae, Bifidobacterium catenulatum, Ruminococcus gauvreauii*, etc., during catabolic pathways, catalyze the phenolics metabolism [47,48]. Therefore, deviation in daily intake of PPs may lead to differences in metabolites of phenolics. Moreover, variations in GM composition are also documented to affect the bioavailability and bioactive effect of PPs and their metabolites [49]. Studies have revealed that PPs can modulate the GM colony by employing antimicrobial activity or prebiotic-like effect against harmful bacteria residing in the gut [50]. In the last decade, the impact of PPs on gut ecology has been studied a lot [51]. Schematic illustrations of sources of PPs and potential GM-associated benefits in humans are depicted in Figure 2. Additionally, the effects of some of the PPs on GM modulation and their effects on metabolic disorders are shown in Table 1.

## 6. Curcumin

**Figure 3:**
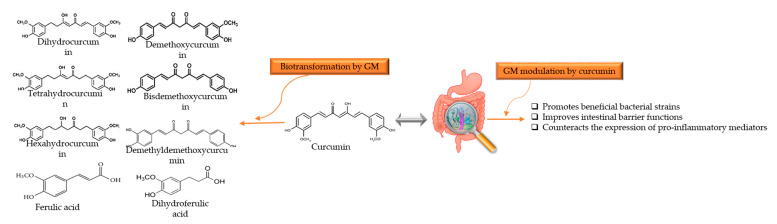
Reciprocal interaction between curcumin and GM. Biotransformation of curcumin occurs due to GM that convert it into several metabolites through pathways like demethylation, reduction, acetylation, hydroxylation, and demethoxylation. These metabolites associated with showing biological activities such as antioxidant, anti-inflammatory, anti-tumoral, and neuroprotective activity. Whereas, GM modulation alters the microbial abundance, diversity, and composition, which also exerts health benefits, indirectly. GM: gut microbiota.

Turmeric, also known as *Curcuma longa L*. belongs to Zingiberaceae (or ginger family) and is a golden-colored spice. Curcumin ((1E,6E)-1,7-bis(4-hydroxy-3-methoxyphenyl)-1,6-heptadiene-3,5-dione) is the principle curcuminoid of turmeric used in traditional medicine to cure various kinds of malady, as well as being a food additive and coloring agent in Asian cuisines and in beverage industries [96]. Hewlings and Kalman [97] stated the beneficial effects of curcumin in the treatment of chronic diseases, such as gastrointestinal, neurological disorders, cardiovascular disease, diabetes, and several types of cancer [98,99,100,101]. Although curcumin has therapeutic properties against many disorders, it has poor bioavailability and low gastrointestinal absorption that is mainly attributed to water insolubility, rapid metabolism, and excretion [101,102]. Enzymes of the large intestine metabolise curcumin, and it is carried out in two phases. In phase-1 metabolism, it yields three metabolites, 1,7-bis(4-hydroxy-3-methoxyphenyl)heptane-3,5-dione (tetrahydrocurcumin), 5-hydroxy-1,7-bis(4-hydroxy-3-methoxyphenyl)-3-heptanone (hexahydrocurcumin), and 1,7-bis(4-hydroxy-3-methoxyphenyl)heptane-3,5-diol (octahydrocurcumin) under reduction. After that, curcumin and its metabolites subject to conjugation through phase-II metabolism to yield sulfate and glucuronide O-conjugated metabolites [103,104]. Transformation not only occurs through enzymes produced by hepatocytes or enterocytes, but also by the enzymes of GM residing in the colon, which can generate many active metabolites [104]. Curcumin metabolites have properties and potency similar to curcumin and exhibit the same physiological and pharmacological properties [105]. It has been stated that curcumin and GM have bidirectional interactions such as GM regulation by curcumin and biotransformation of curcumin by GM [101,105,106]. The reciprocal interaction between curcumin and GM is illustrated in Figure 3. Carmody et al. [107] reported that the biological properties of curcumin depend on the activity of metabolites produced by GM digestion. The curcumin metabolic pathways by GM include reduction, methylation, demethoxylation, hydroxylation, and acetylation, and the main products are 1,7-bis(4-hydroxy-3-methoxyphenyl)heptane-3,5-dione (tetrahydrocurcumin), 3-(4-Hydroxy-3-methoxyphenyl)propanoic acid (dihydroferulic acid), and 1-(4-hydroxy-3-methoxyphenyl)-2-propanol. Furthermore, curcumin can also be metabolized by *Pichia pastoris* into four major metabolites, include 1,7-bis(4-hydroxy-3methoxyphenyl) heptan-3,5-diol, 5-hydroxy-1,7-bis(4-hydroxy-3-methoxyphenyl) heptan-3-one, 5-hydroxy-1,7-bis(4-hydroxyphenyl) heptane-3-one, and 5-hydroxy-7-(4-hydroxy-3-methoxyphenyl)-1-(4-hydroxyphenyl) heptan-3-one [101,103]. Many GM, such as *E. coli*, *E. fergusonii* (ATCC 35469) *Blautia* sp. (*mrg-pmf1*), *Bifidobacterium* (*Bifidobacteria longum* BB536, *Bifidobacteria pseudocatenulaum* G4), *Lactobacillus* (*Lactobacillus casei* and *Lactobacillus acidophilus*), *Enterococcus faecalis* JCM 5803, *Pichia anomala*, and *Bacillus megateriumdcmb-002*, are found biologically relevant in the biotransformation and degradation of curcumin [103,108,109].

### Curcumin and Gut Health

After oral administration, curcumin is distributed in the intestines, and then curcumin exerts its effects on the GM (such as microbial richness, diversity, and composition) [95]. A study conducted by Shen et al. [110] stated that curcumin administration exerts significant effects on GM family such as Bacteroidaceae, Rikenellaceae, and Prevotellaceae. Another study revealed that curcumin administration was effective in weight loss in ovariectomized rats. Moreover, curcumin significantly promoted GM, including *Anaerotruncus*, *Exiguobacterium, Helicobacter, Papillibacter, Pseudomonas, Serratia*, and *Shewanella*. This significance partially reversed the deficiency of estrogen induced by ovariectomy [111]. A very recent study conducted by Al-Saud [112] showed that intragastrical administration of curcumin to type 2 diabetic and obese male albino Wistar rats for 8 weeks (80 mg/kg/day) exhibited anti-obese and anti-diabetic properties, as well as enhancement in the expressions of *GLUT4* gene. Curcumin revealed glucose-lowering effects, dyslipidaemia, decrease in insulin resistance, and malondialdehyde levels in the liver and pancreas. Koboziev et al. [113] supplemented a very high in fat diet with curcumin to mice prone to diet-induced metabolic dysfunction. They found animals to be protected against obesity and osteoarthritis without significant changes in knee cartilage integrity, glucose clearance, and white adipocyte size. Curcumin also ameliorates the intestinal barrier function (by modulating intracellular signaling and the organization of tight junctions) in metabolic diseases, as indicated by a reduced rate of bacterial translocation to the blood, liver, kidneys, and spleen [114]. A study showed that administration of curcumin significantly reduced the Western-diet-induced blood lipopolysaccharide and ameliorated the intestinal barrier [115]. Thus, it can be concluded that curcumin prevents metabolic diseases through a mechanism involved in the regulation of the intestinal barrier.

Many studies suggest that curcumin can actively hinder intestinal inflammation by modulating the homeostasis of the gut-brain axis, and could also exhibit neuroprotective beneficial [104]. Curcumin inhibits lipopolysaccharide-induced nuclear factor-κB (NF-κB) p65 translocation and mitogen-activated protein kinase phosphorylation in dendritic cells that lead to inflammation reduction [116]. As GMD, priming the innate immune system by microbiota, determines a neuroinflammatory response that causes misfolding of neuronal amyloid-β and α-synuclein [117]. Further, curcumin treatment decreases the microbial abundance of cancer-related species like *Prevotella, Coriobacterales,* and *Ruminococcus* [118]. Wu et al. [119] stated that curcumin regulates signaling pathways, such as NF-κB, and nuclear factor erythroid-2-related factor 2, epigenomics/epigenetics pathways of histones modifications, and DNA methylation. These help to exhibit antioxidative and anticancer properties. Another study on curcumin encapsulated with nanoparticles found it effective against colitis as it modulates GM and regulates T-cells [120]. The use of curcumin with randomized clinical trials showed therapeutic effects against ulcerative colitis and Crohn’s disease, but meta-analyses showed controversial results about the therapeutic approach [121]. Tetrahydrocurcumin can decrease the blood glucose level, increase the expression of pancreatic glucagon-like peptide-1, protect islet β cells, and the secretions of insulin in diabetic rats. Furthermore, it restores the intestinal dysbiosis as it lowers the relative abundance of Actinobacteria, Proteobacteria, and Firmicutes/Bacteroidetes ratio [122].

## 7. Quercetin

**Figure 4:**
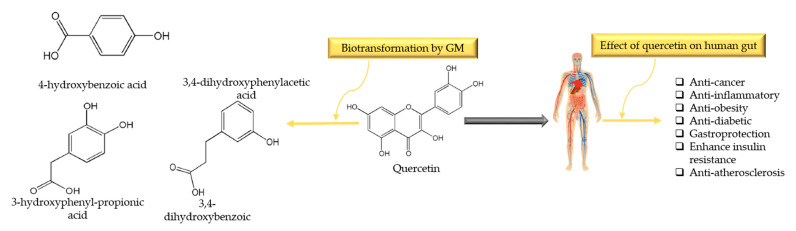
Biotransformation of quercetin into metabolites by gut microbiota (mainly by *Bacteroides fragilis*, *Eubacterium ramulus, C. perfringens*) and their benefits in gut.

Quercetin (3,5,7-trihydroxy-2-(3,4-dihydroxyphenyl)-4Hchromen-4-one) is one of the most common flavonoids present in consumer foods and it belongs to the family of flavonols (myricetin and kaempferol). It is commonly found in green tea, lettuce, radish leaves, cranberry, apple, onion, buckwheat, coriander, lovage, etc. In plants, it is usually bound as ethers or phenolic acids or glycoside/aglycone (with or without linked sugars), etc. Even though it has various forms in nature, the form quercetin-3-*O*-glucoside is found in plants (as sugar moieties like rutinose or rhamnose), which generally acts as a pigment and give color to a multitude of vegetables and fruits [123,124,125]. Daily intake dose of quercetin is ranging from 1 to 250 mg/day [126]. Upon ingestion, quercetin can interact with salivary proteins and form soluble protein–quercetin binary aggregates [127], and in the stomach, quercetin is exposed to the lower pH conditions which may break phenolic acids by bacteria ring fusion [128]. After reaching the small intestine, it is deglycosylated by lactate pholrizin hydrolase (a family of 1 β-glucosidase), yielding quercetin aglycon. About 65–81% of quercetin goes to the liver through the epithelium, where it is metabolized and becomes bioavailable [129]. Complex metabolic reactions in the small intestine and stomach make it bioavailable, with bioavailability reported to be less than 10% [130]. GM transform quercetin into homoprocatechuic acid (3,4-dihydroxyphenylacetic acid), Protocatechuic acid (3,4-dihydroxybenzoic acid), 4-hydroxybenzoic acid, and 3-(3-hydroxyphenyl)propionic acid [131]. Di Pede et al. [132] observed the influence of different formulations on the microbial metabolism of quercetin in a time-dependent manner. It has been documented that *Bacteroides fragilis*, *Eubacterium ramulus*, *Clostridium perfringens*, *Bacteroides JY-6*, *Bifidobacterium B-9*, *Lactobacillus L-2*, and *Streptococcus S-2* are the bacterial strains responsible for the transformation of quercetin into the metabolites [133,134]. Biotransformation of quercetin into metabolites by GM and their benefits in the gut is illustrated in Figure 4.

### Quercetin and Gut Health

It has been mentioned in studies that quercetin has antioxidant, anti-inflammatory, antiviral, anti-obesity, antidepressant properties, as well as preventing cancer, diabetes, asthma, hypertension, and cardiovascular diseases [135,136]. A study reported by Ju et al. [137] stated that quercetin decreases the abundance of *E. coli* and proteobacteria. The genera *Coprococcus_1*, *Anaerovorax, Ruminiclostridium_9, Mucisprillum, Roseburia,* and *Tyzzerella* are also reported to increase in mice after the treatment of quercetin. Another study stated that it promoted the populations of *Bifidobacterium*, *Bacteroides, Clostridia,* and *Lactobacillus* and significantly suppressed *Enterococcus* and *Fusobacterium* [135], thus, it promotes gut homeostasis. A recent study by Lin et al. [138] reported that supplementation of quercetin to mice ameliorated the effects of *Citrobacter rodentium*-induced colitis, terminated the production of pro-inflammatory cytokines including interleukin (IL)-17, IL-6, tumor necrosis factor-α, and enhanced the production of IL-10 in the colon tissues. It also ameliorated the intestinal barrier function with the reduction in the activity of serum diamine oxidase and content of serum D-lactic acid. [135]. A study conducted on diabetic rats suggested that quercetin improved dyslipidemia, decreased serum blood glucose levels, enhanced insulin levels, and decreased oxidative stress injury [139]. Quercetin promotes cell survival and reduces ethanol-induced liver injury, and suppresses autophagic flux in both in vitro and in vivo studies [140,141]. Oral administration of quercetin to rats increased the sexual activity, intromission frequency, mount frequency, sperm count, and motility, and reduced the testicular damage induced by diabetes [142,143]. Intravenous administration of quercetin reported a lowering in blood pressure of hypersensitive rats [144]. Li et al. [145] documented that quercetin can reduce the *Streptococcus suis*-mediated inflammation by inhibiting the suilysin activity both in vitro and in vivo. Other studies on liver disorders suggested that quercetin can protect the liver from ethanol-induced liver fat accumulation and rotenone-induced liver-metabolic imbalances [146,147]. Further, quercetin can decrease lipid peroxidation in both serum and liver tissues and can exert free-radical and ROS-scavenging activity [148,149]. It also enhances superoxide dismutase, glutathione peroxidase, and catalase activities. On the other hand, it decreases lipid peroxidation of bone marrow and spleen tissues [150].

Quercetin is considered beneficial against different types of cancers, including pancreatic cancer, osteosarcoma, breast cancer, cervical cancer, leukemia, colon cancer, gastrointestinal cancer, ovarian cancer, and oral cancer [151]. It has been reported by Zhou et al. [152] that quercetin increased the cytotoxicity of doxorubicin in SW620/Ad300 cells and repressed the transport activity of P-glycoprotein which resulted in overcoming the colon cancer cells’ resistance to chemotherapy by inhibiting solute carrier family 1, member 5 transporter. Quercetin possesses morphological changes, decreases total viability via apoptotic and Bcl-x, and enhances the pro-apoptotic protein Bcl-2 family proteins, such as Bad, Bid, and Bax in AGS human gastrointestinal cancer cells (a human gastric adenocarcinoma cell-line) [153]. Moreover, it can protect intestinal porcine enterocyte cells against H_2_O_2_-induced apoptosis through the hindrance of the mitochondrial apoptosis pathway [154]. Forney et al. [155] revealed that quercetin has anti-inflammatory effects as it ameliorates adipose tissue expansion by reducing the levels of monocyte chemoattractant protein-1 mRNA and serum IL-6 in white adipose tissue of obese mice. Additionally, quercetin ameliorates glucosamine-induced inflammation and apoptosis in human umbilical vein endothelial cells that characterize a model of vascular endothelial injury in the initial stages of atherosclerosis [156].

## 8. Catechins

**Figure 5:**
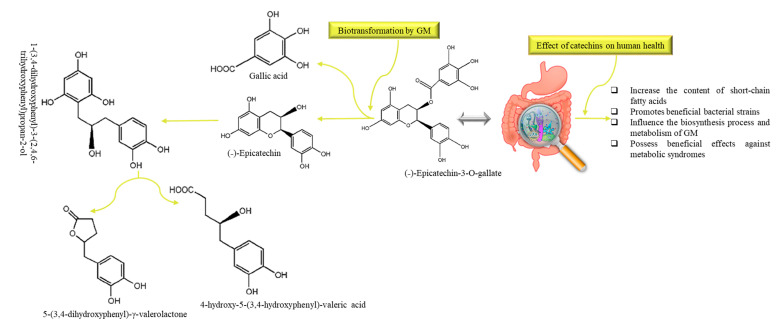
The metabolic pathway and common metabolites of (˗)-Epicatechin-3-*O*-gallate and their modulatory effects on human gut: *Eggerthella lenta* and *Flavonifractor plautii* are mainly responsible for the biotransformation of dietary catechins. GM: gut microbiota.

Catechins are widely distributed in many foods and herbs such as tea, cacaos, apples, persimmons, berries, and grapes [157]. Catechins are one of the main antioxidant agents that are biologically active and present in green tea (*Camellia sinesis*) [158]. Catechins include epigallocatechin-3-gallate (EGCG), epigallocatechin (EGC), epicatechin, epicatechin-3-gallate, gallocatechin gallate (GCG), and gallocatechins, among which EGCG is the most abundant and biologically active [159]. Biotransformation of tea catechins into their metabolites is mainly dependent on the GM. Ingested catechins pass through the small intestine (with very less degradation) and reach the colon where they are metabolized from the microflora, giving rise to both phenylvalerolactones and phenylvaleric acids [160]. Some portions of catechins may undergo extensive phase-I (which include oxidation, reduction and hydrolysis) and phase-II (conjugation) biotransformation in intestinal cells and then form the hepatocytes that result in the rapid release of a series of water-soluble conjugate metabolites such as methyl, glucuronide, and sulfate derivatives. Furthermore, they are converted into small molecular compounds which enter the hepatoenteral circulation or systemic circulation to exert various physiological functions [161]. Kutschera et al. [47] reported two bacterial strains, *Flavonifractor plautii* and *Eggerthella lenta*, which are responsible for the biotransformation of dietary catechins into hydroxyvaleric acid and valerolactones metabolites (illustrated in Figure 5). Particularly, *Eggerthella lenta* convert catechins into 1-(3,4-dihydroxyphenyl)-3-(2,4,6-trihydroxyphenyl)propan-2-ol, and *Flavonifractor plautii* transform it further into 5-(3,4-dihydroxyphenyl)-γ-valerolactone and 4-hydroxy-5-(3,4-hydroxyphenyl)valeric acid [47]. Microbial dihydroxylation reactions could further convert 4-hydroxy-5-(3,4-hydroxyphenyl)valeric acid to 5-(dihydroxyphenyl)-valeric acid and then to 3-hydroxyphenyl-valeric acid [162]. Ultimately, they are absorbed and undergo glucuronidation: 3′-*O*-glucuronide conjugate of (dihydroxyphenyl)-γ-valerolactone is the most abundant valerolactone species in urine after green tea intake [160]. The (dihydroxyphenyl)-γ-valerolactone metabolite showed remarkable antioxidant activity in vitro [163].

### Catechins and Gut Health

Catechins act as anti-inflammatories, antimicrobials, immunomodulators, regulators of ROS production, antioxidants, free radical scavengers, neuroprotective agents, anti-ageing, protectors of the circulatory system and cardiac tissues [164,165]. Catechins can inhibit the growth rate of *H. pylori*, *Staphylococcus aureus*, *E. coli* O157:H7, *Salmonella typhimurium* DT104, *Pseudomonas aeruginosa*, etc. [166]. Mainly, EGCG and GCG inhibit the growth of *Bacteroides*-*Prevotella*, *Clostridium histolyticum*, and *Eubacterium-Clostridium* groups [167]. Liao et al. [168] revealed that tea polyphenols could increase the *Bifidobacteria* and reduce total serum cholesterol and low-density lipoprotein cholesterol levels in mice. Kim et al. [169] treated 3T3-L1 mouse adipocytes with ECG and gallic acid during differentiation adipocytes and found that ECG enhanced adiponectin and uncoupled protein 1 transcription in mature adipocytes. Besides, supplementation of green tea catechins to obese subjects and patients with non-alcoholic fatty liver disease showed a decrease in body fat [170]. Catechins have strong anti-adipogenesis and anti-differentiation effects on mature adipocytes and 3T3-L1 preadipocytes via regulating the cyclic AMP/protein kinase A and C/EBPs/PPARγ/SREBP1C signaling pathways, which can thus exhibit the dual effect of preventing obesity and reducing fat [171]. Other than these, catechins also exhibit antimicrobial activities, as it has been reported that they can inhibit *Salmonella enterica* serovar Typhimurium type III protein secretion and invasion of host cells [172], syntaxin-1 expression [173], and induce endogenous oxidative stress in *E. coli* [174]. Catechin of green tea, EGCG, showed promising results in the inhibition of colon, prostate, lung, pancreatic, intestinal, and stomach cancers [158]. Many studies evidently confirmed the induction of apoptosis and cell cycle arrest by EGCG in colon cancer HCT-116 cells [175]. Additionally, EGCG prevents the activation of the epidermal growth factor receptor, HER2 genes and receptors, and multiple downstream signaling pathways in colon cancer cell lines [176]. Sur et al. [177] documented that EGCG and theaflavin inhibit mouse liver carcinogenesis through modulation of self-renewal Wnt and hedgehog pathways. Grzesik et al. [178] conducted a comparison study among EGCG, epicatechin, epicatechin gallate, gallocatechin gallate, curcumin, and hydrocinnamic acid to check their antioxidant properties. They found that EGCG and epicatechin gallate were the most efficient antioxidants as compared to others. Hence, catechins and their metabolites can regulate intestinal microecological balance by the modulation of the component of intestinal flora.

All of the above-mentioned studies reported protective effects of curcumin, quercetin, and catechins as they can promote beneficial strains by suppressing pathogenic ones. All of the data suggested by these studies cannot be compared because of different formulations, doses, study conditions, time of treatment, route of administration, and interventions being used, and the prebiotic effect of these PPs is probably due to an indirect effect. In most cases, the metabolism of PPs provides a “direct” fitness advantage to gut and GM. Thus, with the support of this data, the ability of curcumin, quercetin, and catechins to positively modulate GM, GMD, and gut may help to understand their therapeutic benefits better. Still, clinical studies are needed to estimate the specific effects of these PPs on the human gut microbiome in patients with metabolic syndromes.

## 9. Bioavailability of Polyphenols

**Figure 6:**
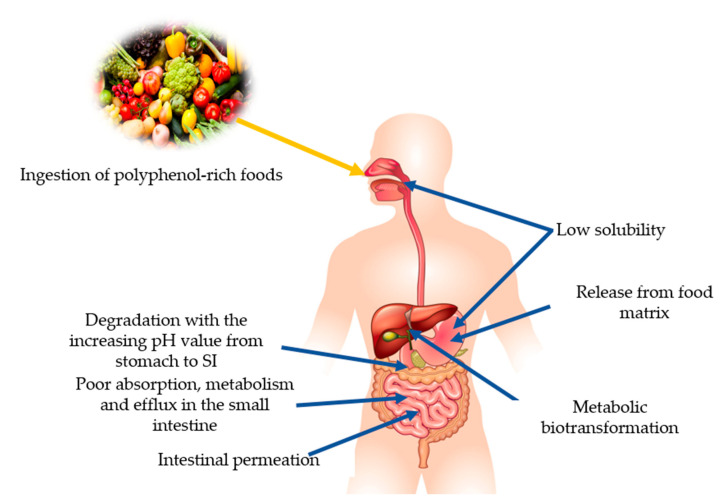
Summary of the factors affecting the bioavailability of polyphenols.

The fraction of a nutrient/non-nutrient that is available to the human body for physiological functions/storage can be referred to as bioavailability [179]. Several factors affect the bioavailability of PPs after ingestion, e.g., low water solubility. After oral administration, the drastic degradation processes start due to the transit in the different organs of the GIT, where the unaffected compounds need to be released from the food matrix to be absorbable. Further, low metabolism in the small intestine and permeation through the intestinal barrier renders them unable to distribute further into the bloodstream [180] (Figure 6). Application of PPs in functional foods and nutraceuticals as a drug molecule is limited due to their low aqueous solubility, inefficient systemic delivery, extensive first-pass metabolism, and poor oral bioavailability [126]. Catechin (especially EGCG), curcumin, and quercetin are the well-known bioactive PPs but they have low bioavailability. To overcome the bioavailability issues and utilization of beneficial properties of PPs, nanoencapsulation can be a promising technique [125]. Substances are encapsulated at the nanoscale with continuous films of coating material that improve the target specificity, stability, solubility, and drug release. They protect the substances during the GIT interaction, intestinal permeation, and increase residence time in intestines. Nano-delivery systems such as lipid-based carriers, polymer nanoparticles, inclusion complexes, micelles, and conjugates-based encapsulation are being used [181]. Other than nanotechnology, microencapsulation is also a new strategy to enhance the availability and their therapeutic effects [180]. Molecules are encapsulated in microscopic capsules that can measure from millimeter to micrometer [182]. Comparatively, the nanoencapsulation technique is considered better than microencapsulation because of the ultrathin layers that help to enhance the mass transport of substances to the islets and reduce the volume of material. Cell and animal models have shown positive results, but studies related to humans are still lacking.

Moreover, Annunziata et al. [183] proposed that fermentation is a natural strategy with minimum environmental impacts to increase the bioavailability of bioactive compounds (e.g., polyphenols) that can help to produce functional foods with higher nutritional values and health-promoting compounds. Fermentation mainly enhances the solubility and converts them into activated form before utilizing them into functional foods. So, the application of one of these strategies might be helpful to overcome metabolic disorders in clinical trials.

## 10. Conclusions and Perspectives

PPs are naturally occurring bioactive compounds which have an important role in human nutrition due to their antioxidative ability and their ability to decrease ROS. Therefore, they are known as promising candidates that can prevent and combat several metabolic syndromes. Regular consumption of PPs can help to ameliorate metabolic disorders such as obesity, diabetes, cancer, and cardio-metabolic disorders. Metabolism of PPs occurs in the intestines; thus, retention of PPs in intestines for a long time can promote beneficial effect on GM, as well as GM enhancing the biological activity of PPs by biotransforming them into active metabolites that help to improve the overall gut health. However, there are still some crucial challenges, such as poor bioavailability, making it difficult to achieve profitable results in in vitro and in vivo studies, which is why their use in functional and nutraceutical foods for therapeutic uses is limited. For this reason, after extraction of PPs, suitable approaches can be used to enhance their bioavailability, e.g., fermentation, micro- and nanoencapsulation. Another challenge is to determine the dosage level, as the concentration of PPs varies in foods, as it is altered by the food preparation methods. PPs interact with GM which influences how many doses are needed to achieve the optimal therapeutic effects. Moreover, without clinical studies, it is quite difficult to use PPs-based treatments, even though animal studies are available.

On the other hand, animal studies are also controversial as they cannot be compared perfectly because of different dosage levels, study conditions, time of treatment, route of administration, and interventions. Besides, the usage of PPs as prebiotics is also a challenge, their role as prebiotics in the human gut varies, depending on the residing probiotic strains; in many studies, the number of patients is also very small. Apart from this, supplementation of probiotics into PPs formulations can be useful to enhance their therapeutic effects synergistically, with improved efficiency. If scientists overcome these issues in both in vitro and in vivo studies, not only metabolic diseases, by promoting the gut homeostasis, but also many associated diseases will possibly get treated.

## Figures and Tables

**Table 1:** Effect of polyphenols on gut microbiota (GM) modulation and their major effects on gut health with applied models.

Polyphenol/Source	GM Modulation	Major Effects	Model	Ref.
Grape polyphenol	Reduce the *Firmicutes* to *Bacteroidetes* ratio, promote the growth of *Akkermansia muciniphila*, *Bifidobacteria,* *Lactobacillus* and *Bacteroides* spp.	Prevent the VacA toxin, a key virulence factor of *Helicobacter pylori*, reduce blood pressure, ameliorate lipid profile and reduce uric acid levels, reduce GMD-mediated and high fat diet-induced metabolic syndrome	In vitro dynamic gastrointestinal simulators, in vivo animal and human study	[52,53,54,55,56,57,58,59]
Green tea polyphenol	Significant effect on Firmicutes and Bacteroidetes community	Reduce weight, prevent the VacA toxin, promote energy conversion by boosting mitochondrial tricarboxylic acid cycle and urea cycle of GM reduce the levels of glucose, triglycerides, and total cholesterol in the blood	In vivo animal and human study	[60,61,62,63,64,65]
Cranberry extract polyphenols	Promote the growth of *Akkermansia*, *Parvibacter,* and *Barnesiella*	Suppress inflammatory bowel diseases, obesity, and insulin resistance, improve glucose homeostasis, fat loss, ameliorate metabolic health during weight loss	In vivo animal and human study	[66,67,68,69]
Procyanidin supplement	Significantly enhance the β-diversity of GM like *Akkermansia* spp. and Bacteroidetes and reduce the Firmicutes-to-Bacteroidetes ratio, and Lachnospiraceae.	Reduce high-fat and high-sugar diet-induced obesity and inflammation, improve metabolic flexibility and increases energy expenditure, beneficial effects on energy metabolism and GM	In vivo animal model	[70,71,72]
Blueberry polyphenols	Alter the composition of Proteobacteria, *Bifidobacterium,* Actinobacteria, *Adlercreutzia*, *Flexispira*, *Prevotella, Helicobacter*, *Deferribacteres*, and *Desulfovibrio*	Reduce inflammation, insulin resistance induced by high-fat high-sucrose diet, ameliorate obesity, chemopreventive effects towards colon cancer through the regulation of angiogenesis, cell proliferation, and apoptosis	In vivo animal and human study	[73,74,75,76]
Orange juice polyphenol	Increase the *Lactobacillus* spp., *Bifidobacterium* spp., and *Parabacteroides* spp., *Bacteroides ovatus, F. prausnitzii*, *Ruminococcus* spp., and *Akkermansia* spp.	Ameliorate low-density lipoprotein-cholesterol, insulin sensitivity, and glucose	In vivo human study	[77,78,79]
Sinapine polyphenol	Supress the Firmicutes-to-Bacteroidetes ratio and enhance the growth of *Blautia, Akkermansiaceae*, *and Lactobacillaceae*	Prevent GMD and obesity-mediated metabolic diseases such as non-alcoholic fatty liver disease and insulin resistance	In vivo animal model	[80]
Sorghum-bran polyphenols	Promote the growth of *Lactobacillus*, *Bifidobacterium,* and stimulate *Prevotella and Roseburia*	Ameliorate gut health, reduce inflammation and oxidative stress in normal and obese subjects	In vitro, in vivo animal model	[81,82,83]
Resveratrol	Suppress the growth of *Enterococcus faecalis,* and enhance the growth of *Bifidobacterium* and *Lactobacillus*	Suppress fat deposition, reduce activities of fecal and host colonic mucosal enzymes such as nitroreductase, α-glucosidase, α-glucoronidase, β-galactosidase, and mucinase	In vivo animal model	[84,85,86]
Quercetin	Reduce *Firmicutes,* Erysipelotrichia and *Bacillus* genus, down-regulation of *Bacillus*, *Eubacterium cylindroides* and Erysipelotrichaceae	Reduce inflammation, insulin resistance induced by high-fat high-sucrose diet	In vivo animal and human study	[87,88]
Polyphenols (from fungi)	Reduce Firmicutes-to-Bacteroidetes ratio and restoration of *Lactobacillus* spp.	Modulate GM composition, reduce inflammation, lead to insulin and body weight reduction	In vivo animal model	[89]
Coffee and Caffeic acid	Increase the metabolic activity of *Bifidobacterium* spp.	Prevent colon cancer metastasis and neoplastic cell transformation by inhibiting TOPK (T-LAK cell-originated protein kinase) and MEK1	In vivo animal and human study	[90,91]
(−)-epigallocatechin-3-gallate	Stimulate growth of *Bacteroides, Christensenellaceae*, and *Bifidobacterium*, reduce the Firmicutes/Bacteroidetes ratio	Prevent GMD, suppress obesity via manipulating intestinal microbiota and low-grade inflammation	In vitro assay in bacterial medium, in vivo animal and human study	[92,93,94]
Quercetin and resveratrol	Reduce the Firmicutes/Bacteroidetes ratio, inhibit the growth of *Bacillus*, *Eubacterium cylindroides* and Erysipelotrichaceae	Reduce high-fat sucrose diet mediated inflammation, GMD, and lipogenesis	In vivo animal model	[87,95]

GM: gut microbiota, GMD: gut microbiota dysbiosis.

## Abbreviations

PPs	Plant polyphenols
GM	Gut microbiota
GMD	Gut microbiota dysbiosis
ROS	Reactive oxygen species
MAE	Microwave-assisted extraction
UAE	Ultrasonic-assisted extraction
HAE	Homogeniser-assisted extraction
RSLDE	Rapid solid-liquid dynamic extraction
GIT	Gastrointestinal tract
EGCG	Epigallocatechin-3-gallate
EGC	Epigallocatechin
GCG	Gallocatechin gallate
IL	Interleukin
NF-κB	nuclear factor-κB
